# Indium Nitride at the 2D Limit

**DOI:** 10.1002/adma.202006660

**Published:** 2020-11-23

**Authors:** Béla Pécz, Giuseppe Nicotra, Filippo Giannazzo, Rositsa Yakimova, Antal Koos, Anelia Kakanakova‐Georgieva

**Affiliations:** ^1^ Centre for Energy Research Institute for Technical Physics and Materials Science Konkoly‐Thege M. út 29–33 Budapest 1121 Hungary; ^2^ Consiglio Nazionale delle Ricerche Istituto per la Microelettronica e Microsistemi Strada VIII, n. 5, Zona Industriale Catania I‐95121 Italy; ^3^ Department of Physics Chemistry and Biology (IFM) Linköping University Linköping 581 83 Sweden

**Keywords:** 2D semiconductors, epitaxial graphene, indium nitride, metal–organic chemical vapor deposition, SiC substrates, wide‐bandgap materials

## Abstract

The properties of 2D InN are predicted to substantially differ from the bulk crystal. The predicted appealing properties relate to strong in‐ and out‐of‐plane excitons, high electron mobility, efficient strain engineering of their electronic and optical properties, and strong application potential in gas sensing. Until now, the realization of 2D InN remained elusive. In this work, the formation of 2D InN and measurements of its bandgap are reported. Bilayer InN is formed between graphene and SiC by an intercalation process in metal–organic chemical vapor deposition (MOCVD). The thickness uniformity of the intercalated structure is investigated by conductive atomic force microscopy (C‐AFM) and the structural properties by atomic resolution transmission electron microscopy (TEM). The coverage of the SiC surface is very high, above 90%, and a major part of the intercalated structure is represented by two sub‐layers of indium (In) bonded to nitrogen (N). Scanning tunneling spectroscopy (STS) measurements give a bandgap value of 2 ± 0.1 eV for the 2D InN. The stabilization of 2D InN with a pragmatic wide bandgap and high lateral uniformity of intercalation is demonstrated.

## Graphical abstract

A bilayer of InN is formed between graphene and SiC by an intercalation process. The coverage of the SiC surface is very high, above 90%. Scanning tunneling spectroscopy (STS) measurements prove a bandgap value of 2 ± 0.1 eV for the 2D InN.

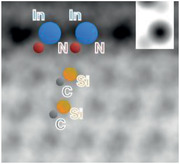

After the discovery of graphene^[^ ^1^ ^]^ and acquiring its extraordinary properties, an extensive research of other 2D materials, including transition metal dichalcogenides and transition metal carbides, was triggered, since most of them could be cleaved from their 3D layered counterparts. By now a lot of new properties of transition metal dichalcogenides have been discovered, which enable designing of not only novel 2D semiconductor materials but 2D electrocatalysts as well.^[^ ^2^ ^]^ 2D group III nitrides are mainly investigated by theoretical modeling predicting a lot of properties which are substantially different from the properties of their 3D crystals. There are a few successful experiments on achieving 2D GaN and 2D AlN,^[^ ^3^, ^4^, ^5^, ^6^ ^]^ which appear as ultrawide bandgap semiconductor materials. The successful formation of 2D GaN was achieved via the intercalation of gallium through bilayer graphene whereby the formation of 2D GaN occurs between the graphene and SiC substrate.^[^ ^3^ ^]^ It is obvious that the synthesis of 2D GaN was a great step resulting in a new structure that cannot be cleaved from the 3D crystal of the same material.^[^ ^4^ ^]^ By similar process of metal–organic chemical vapor deposition (MOCVD), 2D AlN was sandwiched between graphene and (111) Si.^[^ ^5^ ^]^ Clearly, there is a strong value in achieving 2D InN in view of InN being the semiconductor at the low bandgap edge of the group III nitrides.

The number of the published theoretical studies on the structural and electronic properties of 2D AlN and 2D GaN^[^ ^7^ ^]^ by far outcompetes available experimental data; and we can point to the essentially two available papers reporting on the rational achievement of 2D GaN and 2D AlN by MOCVD.^[^ ^3^, ^5^ ^]^

On the other hand, there is only a fragmentary theoretical knowledge about the structural and electronic properties of 2D InN, which is partially ascribed to difficulty in accurate theoretical treatments involving heavy elements such as indium (In).^[^ ^8^, ^9^, ^10^ ^]^ Among 2D group III nitrides, 2D InN is predicted to be of sizable direct bandgap,^[^ ^10^ ^]^ extremely low electron mass, and the highest electron mobility.^[^ ^9^ ^]^ The theoretical studies induced experiments in which the indium intercalation was studied by means of thermal evaporation in an ultrahigh vacuum chamber.^[^ ^11^ ^]^ The experiments showed that the intercalated indium layers are stable at least up to 800 °C.

Here, we report first‐time evidence for 2D InN obtained by MOCVD in the framework of kinetically stabilized processes by intercalation at the interface between SiC and epitaxial graphene. A bandgap of about 2 ± 0.1 eV is extracted from scanning tunneling spectroscopy (STS) measurements which proves a valuable 2D semiconductor material with potential for pragmatic applications.

Epitaxial graphene on SiC was used as a template for the formation of 2D InN. The epitaxial graphene was grown by sublimation method on the Si face of 4H‐SiC.^[^ ^12^ ^]^ Such a structure contains always a graphene buffer layer, which can be transformed into additional graphene layer via hydrogenation resulting in quasi free‐standing epitaxial graphene on SiC.^[^ ^13^, ^14^ ^]^ The MOCVD process of formation of 2D InN was initiated by the delivery of trimethylindium, (CH_3_)_3_In, precursor to graphene. It might be expected that dissociative adsorption of the (CH_3_)_3_In precursor, consequent to the (CH_3_)_3_In/graphene surface reactions, underpins the available pathways for delivery of In adatoms, and their intercalation. This expectation is substantiated by previous ab initio molecular dynamics simulations of surface reactions of trimethylaluminum precursor with graphene and being of support to the conceptual understanding of dissociative adsorption and intercalation phenomena under MOCVD conditions in general.^[^ ^15^ ^]^

The intercalated structures were first characterized by conductive atomic force microscopy (C‐AFM). The front‐to‐back biasing configuration depicted in **Figure** **1**a was employed to probe the current injection from a nanoscale Pt tip to the graphene/InN/SiC heterostructure.

**Figure 1:**
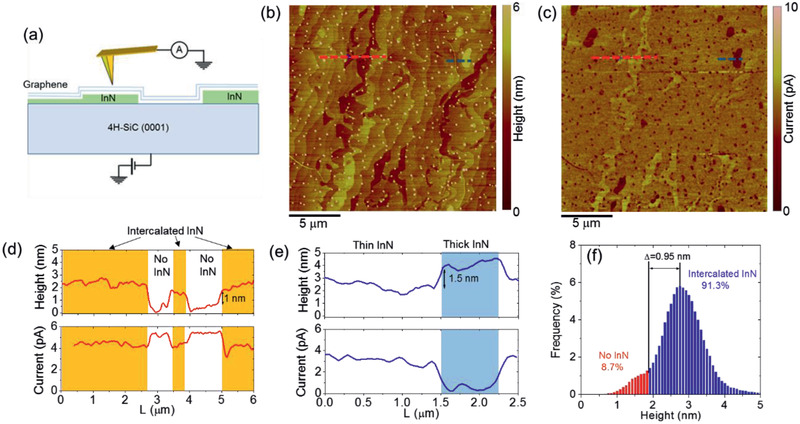
a) Schematic representation of the C‐AFM setup for front‐to‐back current measurements. b) Surface morphology and c) current map collected on a 20 µm × 20 µm sample area. d) Height and current line‐scans extracted along the red dashed lines in the morphology and current maps. Regions without InN can be identified from the higher value of the vertically injected current. e) Height and current line‐scans extracted along the blue dashed lines in the morphology and current maps. A region with a thicker InN island can be identified by the reduced current as compared to the surrounding thin InN. f) Histogram of the height distribution extracted from the morphological image, from which >90% of InN‐intercalated surface area was deduced.

Figure 1b,c shows a representative morphology and the corresponding current map collected on a large sample area (20 µm × 20 µm), in order to obtain statistically relevant information on the uniformity of InN intercalation. Looking at the morphology, the presence of micrometer wide terraces separated by sub‐nanometer high steps can be observed, which is reminiscent of the 4H‐SiC substrate topography. No features related to these substrate‐related steps were observed in the current map in Figure 1c, which showed a uniform signal on most of the probed area, suggesting a homogeneous InN intercalation between graphene and 4H‐SiC. A higher current level than the average value can be observed only on some terraces that were associated to regions without InN intercalation by comparison with the morphology map. Furthermore, small islands with reduced current than the surrounding regions can be noticed in Figure 1c, which are due to thicker intercalated InN layers. Figure 1d shows a representative height and current line‐scans extracted from a region with and without InN intercalation (see red dashed lines). Since the biased metal tip experiences a different electrostatic interaction with graphene in the InN‐intercalated and not intercalated areas, the measured step height of ≈1 nm between these regions does not exactly represent the real thickness of the intercalated InN layer, but it can be considered as the upper limit for this value. The height and current line‐scans in a region comprising both the thin InN and a thick InN island (see blue dashed line) are also reported in Figure 1e, showing a step height of ≈1.5 nm between the two regions. Finally, the histogram of the height distribution extracted from the morphological image is reported in Figure 1f, from which >90% of InN‐intercalated surface area was deduced.

The properties of the intercalated InN were further investigated with atomic resolution for what high resolution transmission electron microscopy was performed to define details of the structures. The TEM lamella was prepared by ion milling, the details are given in the Experimental Section. **Figure** **2**a shows a representative high‐angle annular dark‐field (HAADF) image of intercalated InN taken in scanning transmission electron microscopy (STEM) mode at 200 keV. The image shows two sub‐layers of In with high‐intensity *Z* contrast underneath bilayer of graphene.

**Figure 2:**
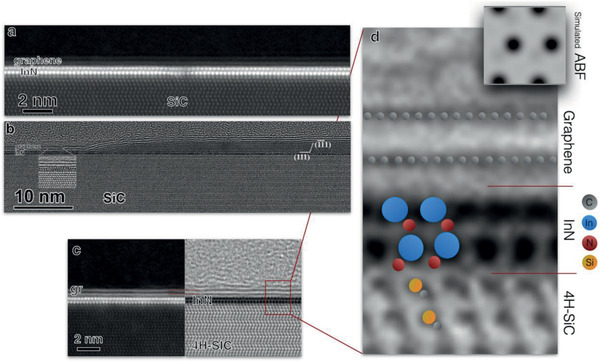
Images taken for the bilayer of InN: a) STEM HAADF image of graphene/SiC template intercalated with trimethylindium and ammonia. Two sub‐layers of intercalated In with high‐intensity *Z* contrast underneath bilayer of graphene are clearly seen. b) HREM image of the same specimen taken in TEM mode at 200 keV. c) ABF/HAADF STEM overview image of the intercalated InN on SiC. d) Magnified from the same image with an insert of simulated image calculated by JEMS software.

The first study of the grown samples at 200 keV did confirm the indium and nitrogen content of the thin layer; especially the indium appears with bright contrast in the HAADF image of Figure 2a. Moreover, the EDS analysis confirmed the presence of nitrogen in the intercalated bilayer structure despite the difficult detection, on what we give details in the Supporting Information.

Several TEM lamellas were prepared from the same sample and investigated which also included taking overview images. Based on that study, it is concluded that the regions of intercalation are mainly represented by bilayer InN. There are regions, less than 10% of the surface, where intercalation did not occur (this is in agreement with the CAFM results).

And finally, there are occasionally a few nm thick regions of InN as well, like it is shown on the right part of Figure 2b. With reference to this region of intercalated InN, the measured lattice spacing is 0.285 nm. This value corresponds to the (111) lattice spacing of cubic InN. Cubic InN itself gains attention by being intentionally grown in the form of a few nm thick layer and applied for the fabrication of field‐effect transistors.^[^ ^16^ ^]^ Growing InN via RF molecular beam epitaxy (MBE) Ishimaru and coworkers found that the growth turns to 3D mode after the 3^rd^ layer is grown despite the layer by layer growth of MBE.^[^ ^17^ ^]^

It is obvious that this few nm thick region can be indexed as cubic InN, two of the (111) type planes are marked in the image. The other lattice spacings in the 3D part can be measured on the HREM image directly and given at the marked region also 0.285 nm along the two <111> type directions, what gives perfectly the appropriate values for the cubic InN (with *a* = 0.49 nm). The measured angles are in good agreement to the fcc cubic InN. We have to note that in some other regions little bit lower d values are measured; however, this few nm thick cubic layer is not a perfect crystal and most probably is strained.

Figure 2c illustrates the sub‐layers of indium (In) and nitrogen (N) atoms. Although indium atoms were already imaged very easily with high brightness on the HAADF images, light atoms such as N, are not visible. To eliminate this limitation annular bright‐field (ABF) STEM method was applied by collecting the electrons within a ring‐shaped circumference from 11 to 24 mrad, thus, cutting the central part of the bright‐field disk out. The ABF method enables to effectively visualize atomic columns composed of light atoms. On the other hand, the increased scattering at high angles outside of the ABF detector of the heavy atomic columns image them as large dark spots. On the overall, the ABF‐STEM method allows to image both low and high mass atoms simultaneously. The image in Figure 2c is an atomic resolution overview from which the next one (Figure 2d) is magnified. In the second image the indium atoms are the large very dark discs, while the nitrogen is also seen with grey contrast and some of the nitrogen atoms are marked by red circles in the image.

The characterization was repeated at 60 keV in a probe‐corrected microscope equipped with an EELS spectrometer. The investigation confirmed the ultrathin regions of bilayer InN together with the detection of the occasional thicker regions of intercalated InN. (Examples are shown in the Supporting Information). On a relatively thick region of 3 nm InN the nitrogen peak, what is very difficult to detect especially without tilting the specimen to the EDS detector, was separated from the close indium Mα peak and the spectrum is shown in the Supporting Information.

X‐ray photoelectron spectroscopy (XPS) measurements were also carried out on the sample and confirmed the presence of the In 3d and N 1s peaks in the InN intercalated sample, details are given in the Supporting Information. However, XPS is not able to reveal further details due to the inherent lack of spatial resolution of this technique. For this reason, atomic‐resolution STEM/EELS have been adopted as methods of choice to get information on the structure and chemical bonding of intercalated InN.

EELS measurements could confirm that the bilayer InN does not contain oxygen. The spectrum recorded, quantification results and other details are given in the Supporting information. In **Figure** **3** the EELS spectrum of the bilayer InN is plotted together with the spectrum of In_2_O_3_ taken from the Gatan EELS Reference Atlas.^[^ ^18^ ^]^ The experimentally measured EELS spectrum is background subtracted. Furthermore 78 EELS spectra are integrated, all of them taken on two parallel lines of InN below the graphene and above SiC, and finally improved by performing principal component analysis (PCA) on the entire hyperspectral EELS data set by applying the method described by G. Lucas et al., Ref. ^[^ ^19^ ^]^. It is worth comparing the obtained EELS spectrum of the bilayer InN to the EELS spectrum with its fine structure taken from InN and reported earlier by K. A. Mkhoyan and co‐workers.^[^ ^20^ ^]^ In that work,^[^ ^20^ ^]^ the fine structure of the N K edge in InN is presented together with the calculated nitrogen N 2p partial density of states. The peaks in the fine structure of nitrogen K edge can be recognized in our spectrum as well and are in good agreement with the findings of Ref. ^[^ ^20^ ^]^ The above EELS results prove that pure InN is formed and stabilized in our 2D InN.

**Figure 3:**
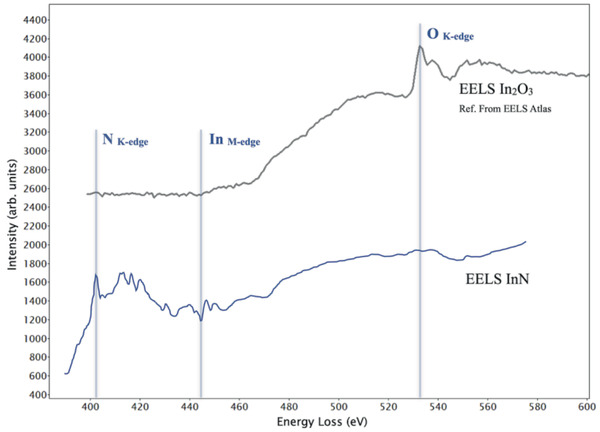
EELS study of the intercalated InN on SiC showing the measured EELS spectrum (background subtracted) and as a comparison the spectrum of In_2_O_3_ taken from the Gatan reference library.^[^ ^18^ ^]^

Further EELS measurement focused on the determination of the bandgap of the bilayer InN, but due to the relatively low bandgap it could not be successful. However, the EELS spectrum taken at 60 keV was analyzed and after deconvolution a peak of 11.7 eV could be observed, which is characteristic of graphene. At the position of InN another peak of 10.88 eV was observed, what we interpret as the surface plasmon peak of InN (details are given in the Supporting Information).

Measurements to determine the bandgap of the intercalated InN were carried out by scanning tunneling microscopy (STM) operating in the STS mode.^[^ ^21^ ^]^ The employed experimental setup is schematically depicted in **Figure** **4**a. In order to directly probe the intercalated 2D InN, the overlying graphene sheets were initially burned out by applying a high bias to the tip. Immediately after, *I–V* curves showing the presence of a bandgap in the density of states were observed. Each STS measurement was repeated 10 times at the same positions of the STM tip, after applying a bias to locally burn the graphene underneath the tip, and the average of 10 *I–V* curves was calculated. The *I–V* curves were stable in time. Sets of measurements were also repeated at more than 10 different positions, and the *I–V* curves were reproducible. Figure 4b (solid line) shows a characteristic STS curve from which the forbidden bandgap of 2 ± 0.1 eV was extracted for the 2D InN (the statistics of the measurement are shown in the Supporting Information). This value differs a lot from the value of 0.7 eV for the bulk (or thick) InN crystals, however it is reasonable to observe a larger bandgap due to the quantum confinement as previously reported in the case of 2D GaN.^[^ ^3^ ^]^

**Figure 4:**
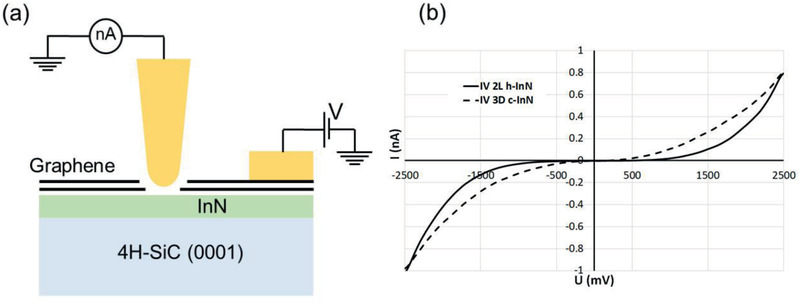
a) Schematic representation of the setup for STS measurements on 2D InN and b) *I–V* characteristic of the bilayer InN (2L h‐InN) and the thicker region (3D c‐InN) respectively.

It is worth pointing out that the bandgap of 2D InN with a value measuring 2 ± 0.1 eV, as extracted from the STS measurements, agrees with the predicted value of 1.99 eV for buckled bilayer InN.^[^ ^10^ ^]^ Buckled bilayer InN with hydrogen passivation has been ascribed by modeling as one of the most stable stacking patterns of 2D InN,^[^ ^10^ ^]^ while this type of stacking pattern can be recognized in the image of Figure 2d.

To further understand the properties of 2D InN, the case of 2D GaN can be considered. As to 2D GaN, very large bandgap energy values were reported ranging from nearly 5.0 eV^[^ ^3^ ^]^ to within (4.18–4.65) eV depending on the particular intercalated structure.^[^ ^22^ ^]^ When a large area 2D GaN was formed on liquid gallium surfaces via a surface confined nitridation reaction, the bandgap energy value was measured as 3.8 eV.^[^ ^23^ ^]^ The difference in the bandgap energy values can be perceived in view of the different methods of formation and obtained nanostructures. The experimental evidence of larger bandgap energy values compared to the bandgap energy value of 3D GaN present a signature of quantum confinement effect in the 2D limit in the above cases. The modeling calculations give good agreement to the experiments when the nanostructure is known, like in the case of “buckled” 2D GaN.^[^ ^3^ ^]^

Ref. ^[^ ^3^ ^]^ presents a plot of bandgap energy as a function of number of buckled layers for the case of 2D InN as well. This plot indicates that the value of the bandgap energy can range from about 3.0 eV for a single layer to nearly 1.0 eV for five layers. Noticeably, the value of the bandgap energy for buckled bilayer InN corresponds to about 2.0 eV, which is in correlation with the STS results for bilayer InN, (Figure 4b). The same figure also contains the *I–V* curve (dashed line) corresponding to the thicker regions of intercalated InN observed in the HREM image in Figure 2b and (marked by 3D c‐InN) from which a lower bandgap of ≈1 eV has been extracted. These regions represent a low percentage (<10%) of the intercalated area, as deduced by the C‐AFM current map in Figure 1c. The most important result of the STS measurements is the value of the bandgap energy for buckled bilayer InN corresponding to 2.0 eV.

In summary, we reported the formation of 2D InN with very high coverage using graphene/SiC templates in MOCVD processes. Most of the intercalated 2D material is represented by bilayer InN, which is consistent with findings from an available theoretical modeling of the most stable stacking patterns of 2D InN. The bandgap of 2D InN is determined to measure a value of 2 eV, which agrees with the theoretical predictions as well. Thus, InN is revealed as an achievable semiconductor at the 2D limit with a pragmatic bandgap, what opens new applications. Moreover, the MOCVD method provides a more general route for the synthesis of 2D group III nitrides.

## Experimental Section

### Epitaxial Graphene on SiC

The used templates, that is, the epitaxial graphene on 4H‐SiC, were formed by high‐temperature sublimation technique (also known as thermal decomposition of SiC)^[^ ^12^, ^24^ ^]^ Si‐face SiC was used to grow graphene which is epitaxial in nature and grows predominantly as one monolayer (ML) graphene about 0.35 nm thick. It is spread as a carpet over the SiC substrates covering continuously the whole surface without disruption at the steps, the latter being typical for these substrates. The consequence is that bilayer graphene growth is promoted on the steps resulting in small patches. Another specific feature of the graphene templates used here is that as grown on the Si‐face they contain a buffer layer (zero‐layer graphene) which is an interfacial layer between SiC substrate and the 1st graphene layer, that is, it is preceding the formation of graphene. The typical size of the samples was 7 × 7 mm^2^.

### Metal–Organic Chemical Vapor Deposition (MOCVD)

The MOCVD processes were performed in a horizontal‐type hot‐wall MOCVD reactor (GR508GFR AIXTRON) which is designed for the research and development of group III nitrides of semiconductor quality.^[^ ^25^ ^]^ The epitaxial graphene was heated in molecular hydrogen at a gas‐flow rate of 25 slm up to the temperature of 700 °C. The reactor was operated at a pressure of 200 mbar. Trimethylindium, (CH_3_)_3_In, and ammonia, NH_3_, were employed as precursors at a gas‐flow rate of 0.875 sccm and 2 slm, respectively. The scheme of precursor delivery involved 3 cycles in total each of about 3 min and consisting of alternating flows of (CH_3_)_3_In with NH_3_ followed by an extra time of 10 min of their joint delivery. A purge flow of H_2_ separated the alternating flows of (CH_3_)_3_In with NH_3_ in each cycle. The flow of H_2_ and NH_3_ continued during the cooling down stage of the overall MOCVD process.

### Atomic Force Microscopy (AFM) and Scanning Tunneling Microscopy/Spectroscopy (STM/STS)

The morphology of the samples was evaluated by tapping mode AFM using Si probes and a DI3100 equipment with a NanoScope V controller. C‐AFM was further employed to probe vertical current injection through the epitaxial graphene/InN/SiC interface. Current mapping was carried out using Pt‐coated Si tips with 5 nm curvature radius. The STM/STS investigation was carried out at room temperature with a Bruker NanoScope 8 scanning probe microscope using PtIr (80−20%) tip.

### Transmission Electron Microscopy (TEM)

TEM was performed to define details of the structure of the ultrathin films. Transparent TEM lamella was cut by focused ion beam (FIB) in which the energy of Ga ions was reduced to 2 keV in a dual beam FEI SCIOS2 equipment. TEM was carried out in a FEI THEMIS 200 microscope equipped with an image corrector at 200 keV. Then STEM was carried out in a probe corrected JEOL ARM 200F microscope also equipped with an electron energy loss spectrometer (EELS), Gatan Image Filter Quantum. A cold emission field emission gun (FEG) electron source provides an energy resolution of 0.3 eV in EELS. For this type of investigation first the e‐beam energy was chosen for 60 keV in order to reduce beam damage especially in the case of sensitive materials such as graphene layers over a long acquisition time. SI (spectral imaging) An EELS data set was recorded at a very short exposure time for the high loss of 2 ms per pixel and summing them over 10 cycles after prior zero loss alignment, the total SI EELS data set acquisition time was 1 min. The SNR (signal to noise ratio) of the EELS spectrum was improved as follows: The acquisition of a big spectral imaging EELS data set over the SiC/InN/graphene interface was done. The data size was 8 × 38 × 2048 pixels with each 3D pixel size of 0.38 nm × 0.38 nm × 0.25 eV. Each 3D pixel, nominally voxel, contains the sum of 10 spectra acquired in an energy range of 512 eV and dispersion 0.25 eV. The spectrum presented in Figure 3c comes from the integration of 2 × 39 voxels acquired over InN bilayer, in a region below the graphene bilayer, and above the SiC substrate.

Once acquired, the entire spectrum imaging EELS data set has been firstly cleaned of spike noise, then multiple scattering deconvolution is applied. SNR has been finally improved by performing PCA on the entire data set by the recombination of most significant components, as in the method described by G. Lucas et al., ref. ^[^ ^19^ ^]^.

## Conflict of Interest

The authors declare no conflict of interest.

## Author Contributions

A.K.‐G. designed the experiments and developed the MOCVD processes. R.Y. prepared the epitaxial graphene on SiC. F.G. carried out the C‐AFM measurements. A.K. determined the bandgap by STS. B.P. took TEM images while STEM investigation and analytical measurements in STEM were carried out by G.N. B.P., A.K.‐G, and F.G. wrote the paper. All authors discussed the results and commented on the manuscript.

## Supporting information

Supporting Information
