# Supplementary material for: Indium Nitride at the 2D Limit
Source: Adv Mater. 2020 Nov 23;33(1):2006660. doi: 10.1002/adma.202006660 (PMC11468865; doi:10.1002/adma.202006660)
Supplement: Supplementary file 1 — Supporting Information [file ADMA-33-2006660-s001.pdf]

© 2020 Wiley-VCH GmbH

ADVANCED MATERIALS

Supporting Information

for *Adv. Mater.*, DOI: 10.1002/adma.202006660

Indium Nitride at the 2D Limit

Béla Pécz, Giuseppe Nicotra,* Filippo Giannazzo, Rositsa Yakimova, Antal Koos, and Anelia Kakanakova-Georgieva**

Supporting Information

Indium Nitride at the 2D Limit

Béla Pécz, Giuseppe Nicotra*, Filippo Giannazzo, Rositsa Yakimova, Antal Koos and Anelia Kakanakova-Georgieva**

1) EDS analysis taken at 60 keV:

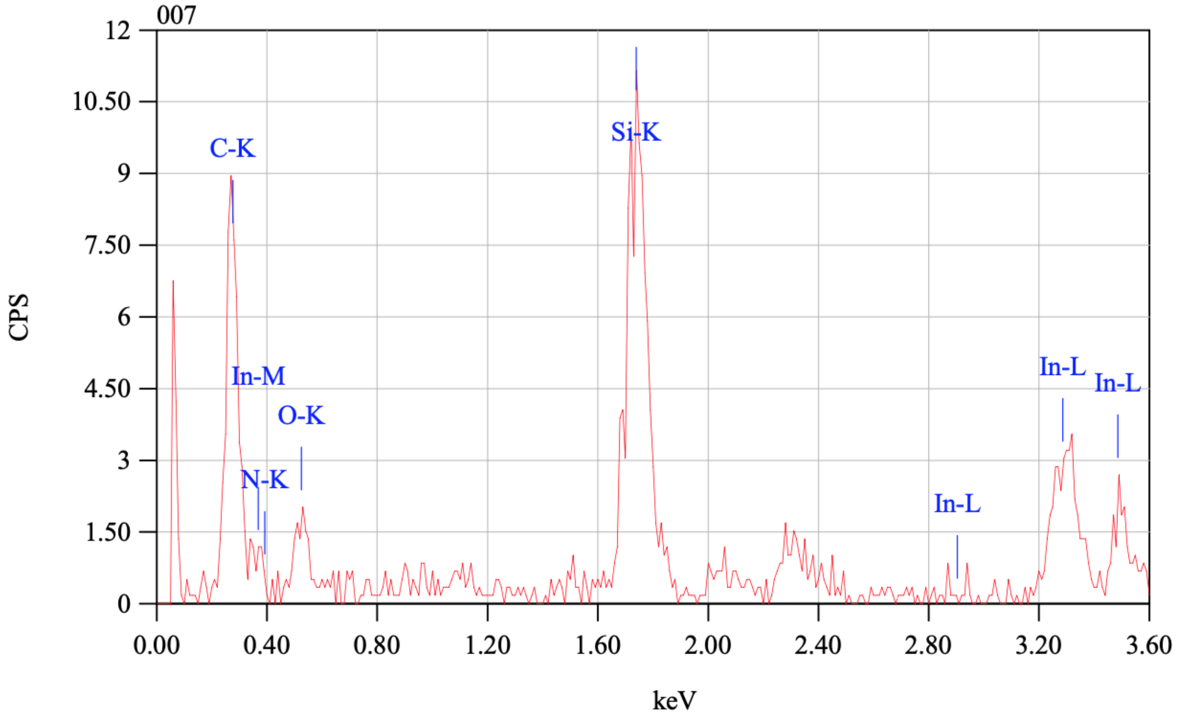

Fig.S1: EDS spectrum of the InN layer showing the "main" In Kα peak at 3.286 eV, the nitrogen peak at 392 eV and the In Mα peak at 368 eV. Also some oxygen is seen.

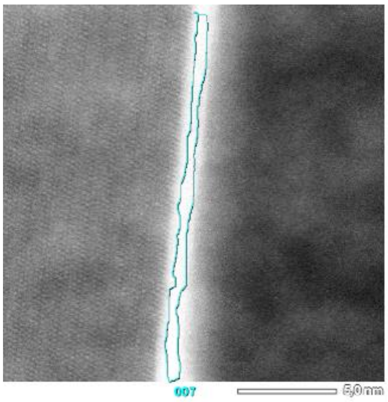

a)

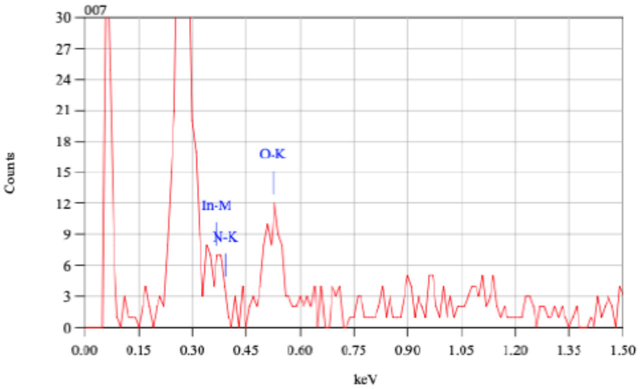

b)

Fig.S2.a) Thin InN layer spectrum image

Fig.S2.b) EDS spectrum extracted from the area marked in the previous image.

Thin Film Standardless Standardless Quantitative Analysis
Fitting Coefficient : 0.7176

Element	(keV)	Mass%	Counts	Sigma	Atom%	Compound	Mass%	Cation	K
N K*	0.392	11.30	110.19	0.14	44.42				0.2852
O K*	0.525	4.40	57.61	0.22	15.15				0.2125
In L (Ref.)	3.286	84.30	234.38	0.09	40.42				1.0000
Total		100.00			100.00				

The quantitative analysis of the extracted EDS spectrum was carried out and gave the above values. When we suppose that half of the layer is indium, then we get an about 12 at% oxygen content in the thin InN layer. However, EDS does not provide information so far loalized as EELS, therefore see the EELS results in the main text of the manuscript.

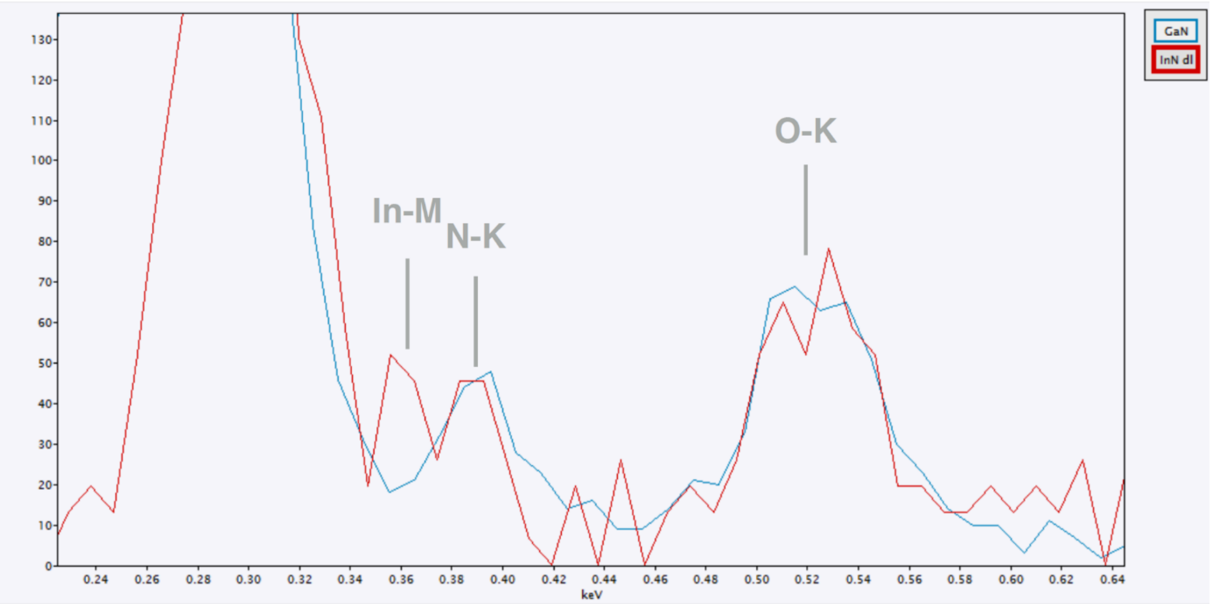

Fig. S3: Comparison of GaN bilayer case (not discussed in this paper) and InN in the EDS spectra.

The above figure clearly shows that we can detect indium and nitrogen as well in the case of InN thin layers.

2) EELS analysis taken at 60 keV, JEOL ARM probe corrected and Gatan EELS Quantum:

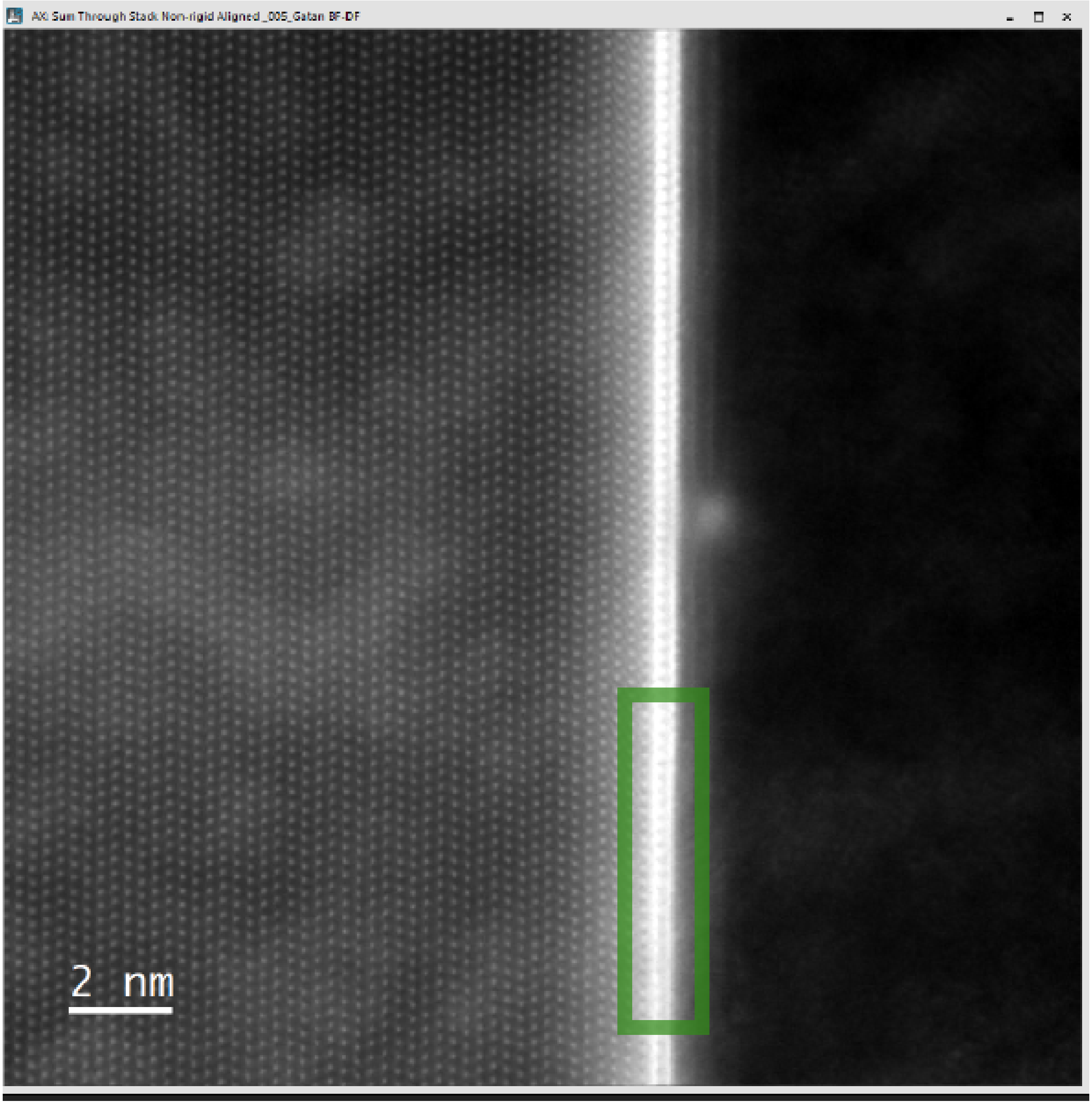

Fig. S4 HAADF image showing the bilayer of InN and the box is the area where the next spectrum is taken from.

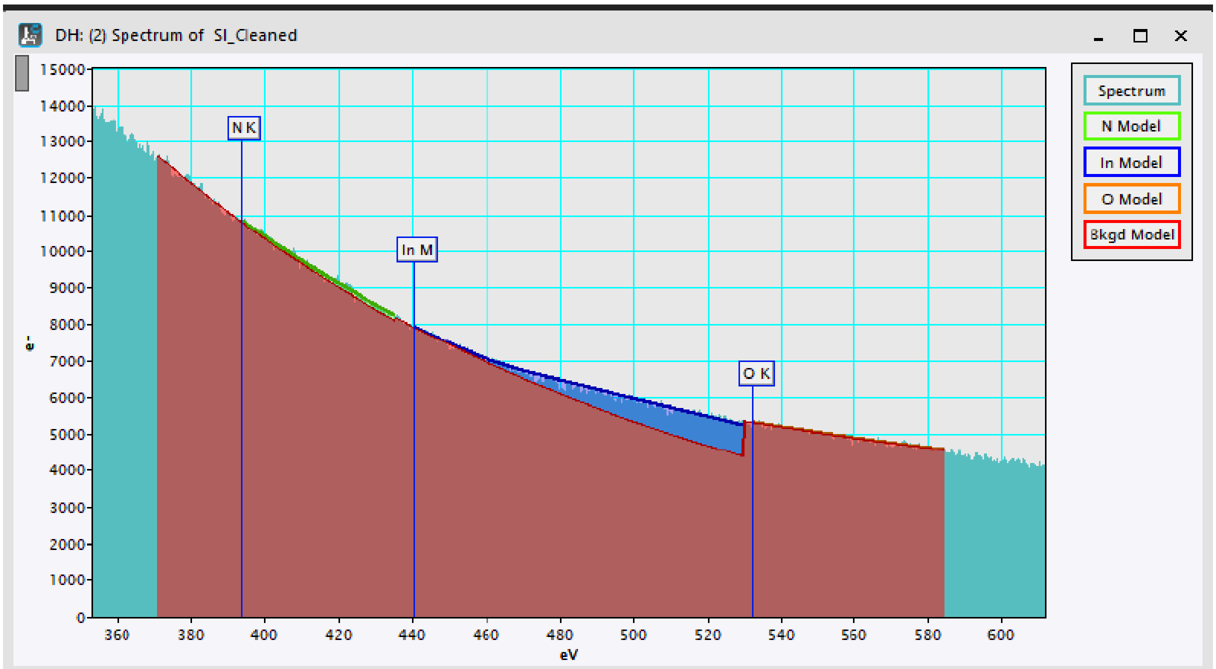

Fig. S5 The EELS spectrum taken from the green box region marked on Fig. S4

Composition Results

Element	Shell	Signal (e-)	Comp. (at.%)	Rel. comp. (/In)
N	K	16.5e+03 ± 1.3e+03	47 ± 6	0.89
In	M	149.3e+03 ± 1.5e+03	53 ± 6	1.00
O	K	0 ± 1.0e+03	0.00 ± 0.04	0.00

Fig. S6 The results of the composition analysis showing InN and no oxygen.

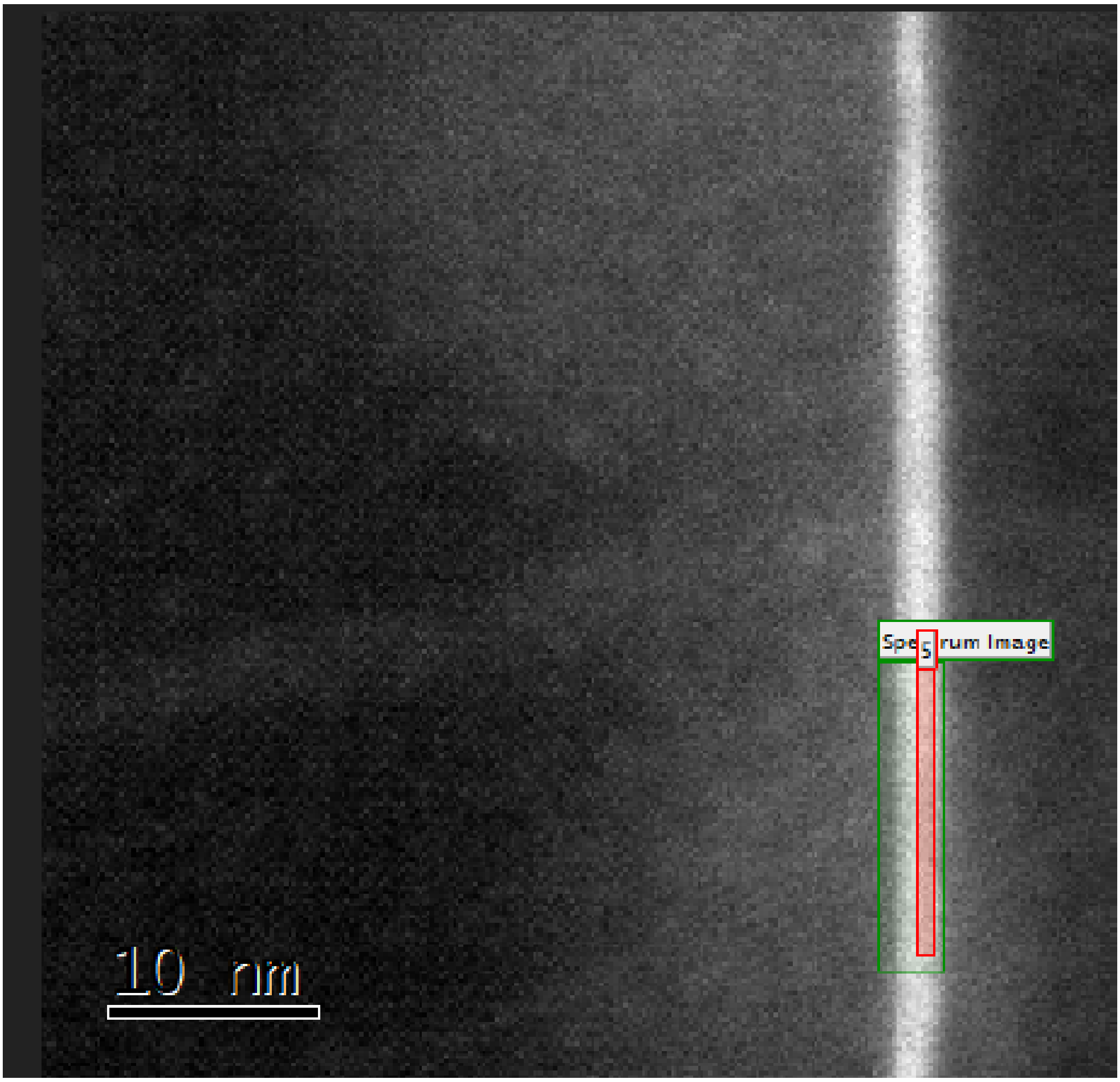

Fig. S7 The red box shows the area, where the EELS spectrum shown in Fig. 4d was extracted from the spectrum image.

Low loss spectrum:

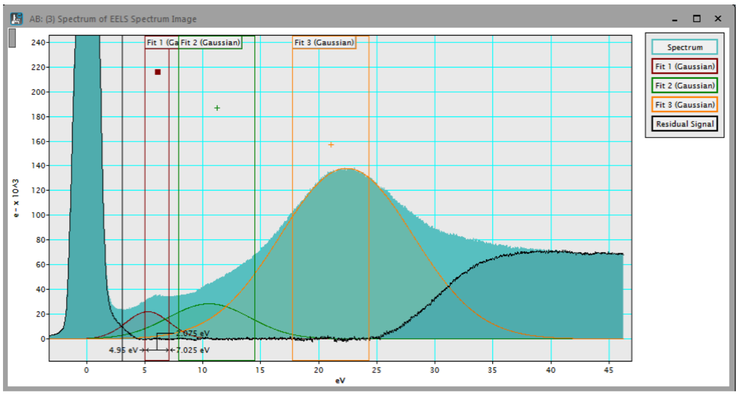

Fig. S8: Low loss part of the EELS spectrum taken on the InN layer

The above experimental spectrum is evaluated by deconvolution. The peak at 22 eV belongs to the amorphous carbon (intentionally deposited to our specimen), while the deconvoluted peak at 5 eV belongs clearly to the graphene π peak. We know that the InN plasmons peak ought to be found at 15.4 eV, while we observe here the surface plasmon peak of InN at 10.88 eV. Reference: Frederick Wooten: Optical Properties of solids, Academic Press, 1972, Chapter 9, equation 9.34.
This is also an indication for the 2D InN formation.

3) STS measurements

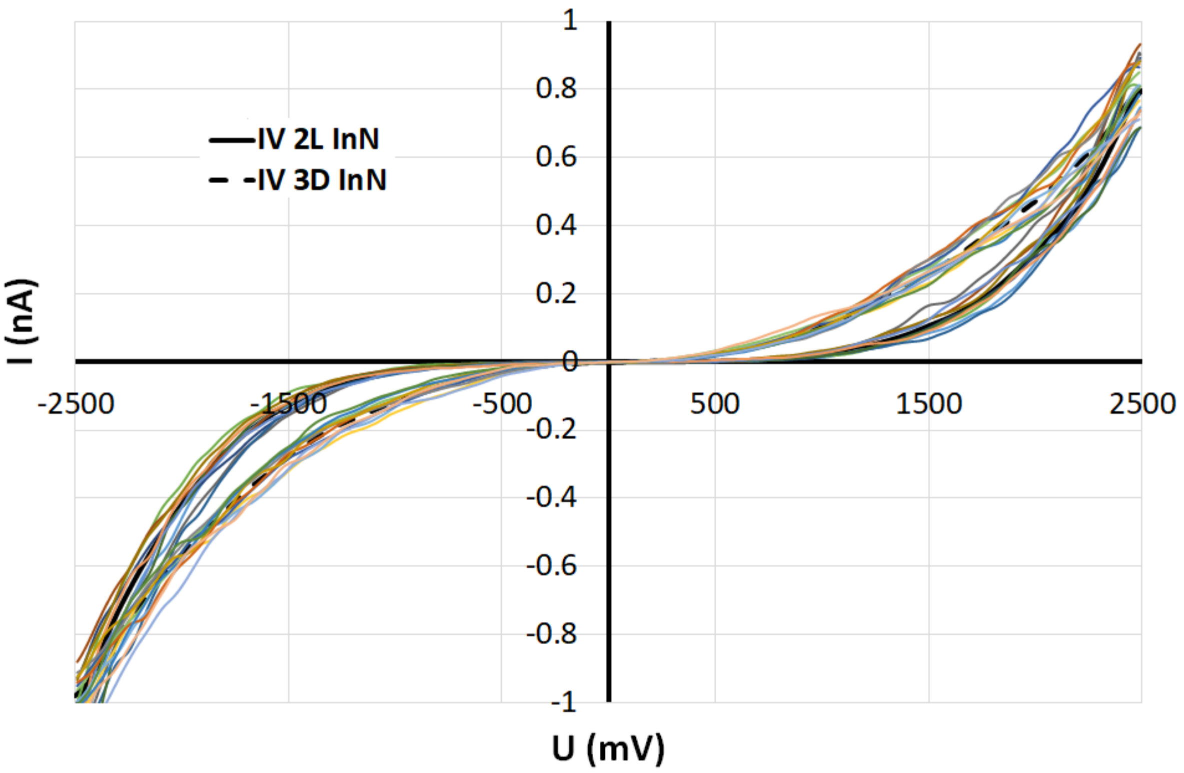

Fig. S9: Statistics of the measured I-V curves for the bilayer InN and for the 3D regions as well measured by STS. The two curves are separated clearly.

4) XPS analysis:

XPS measurements have been carried out both on the EG/SiC sample intercalated with InN by NH_3/TMIn (ID S#3) and on two reference EG samples subjected to annealing in H_2 (ID S#2) and NH_3 (ID S#3) gas, respectively. The existing InN sample was covered intentionally by carbon, that is advantageous for the TEM sample preparation. That specimen was used for XPS as well.

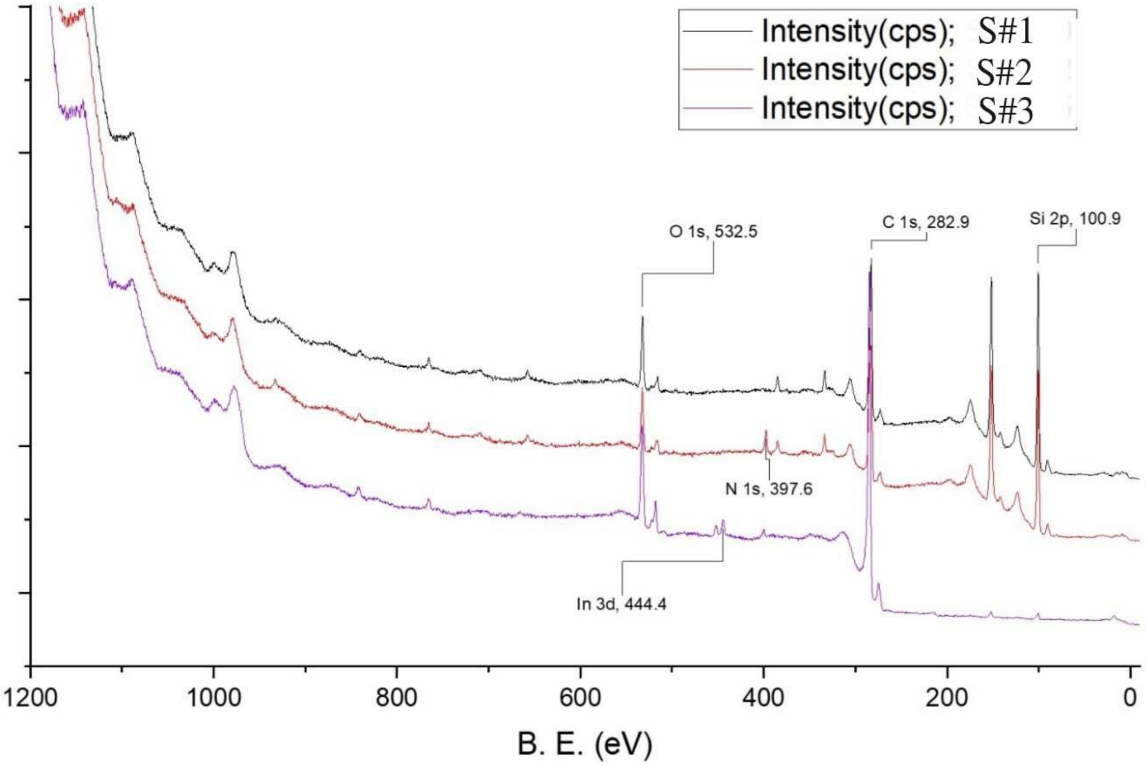

Fig. S10. The figure above shows survey XPS spectra on the three samples with ID numbers as indicated above.

The presence of the In 3d and N 1s peaks is clearly visible in the InN intercalated sample, whereas the N 1s peak only is visible in the sample treated with NH_3. Noteworthy, the presence of the O 1s peak is observed in all the three samples, which reveals the oxidation of some regions, probably after exposure to air. However, XPS is not able to reveal if the intercalated InN is oxidized, due to the inherent lack of spatial resolution of this technique. For this reason, atomic resolution STEM/EELS have been adopted as methods of choice to get information on the structure and chemical bonding of intercalated InN.
